# The effect of training the fathers to support their wives on stress and self-efficacy in mothers of premature newborns hospitalized in NICU: a quasi-experimental study

**DOI:** 10.1186/s12884-022-04413-8

**Published:** 2022-02-04

**Authors:** Zahra Hadian Shirazi, Hamed Ghasemloo, Seyyed Mostajab Razavinejad, Nasrin Sharifi, Shahpar Bagheri

**Affiliations:** 1 Department of Nursing, School of Nursing and Midwifery, Community Based Psychiatric Care Research Center, Shiraz University of Medical Sciences, Shiraz, Iran; 2 Student Research Committee, Department of Nursing, School of Nursing and Midwifery, Shiraz University of Medical Sciences, Shiraz, Iran; 3 Department of Pediatrics, School of Medicine, Neonatal Research Center, Nemazee Teaching Hospital, Shiraz University of Medical Sciences, Shiraz, Iran

**Keywords:** Fathers, Mothers, Neonatal intensive care units, Self-efficacy, Stress, Training

## Abstract

**Background:**

The birth of premature newborns and their separation from family due to their hospitalization in the Neonatal Intensive Care Unit (NICU) cause stress in the parents, especially mothers. We conducted this study aimed to evaluate whether training the fathers to support their wives impacts premature newborn mothers’ stress and self-efficacy or not?

**Methods:**

A quasi-experimental (before-after study) including one experimental and control group was used. Data were collected from Seventy-five parents with newborns hospitalized in NICU (*n* = 30) in the intervention and (*n* = 45) in usual care groups. Settings were the NICUs of the two international, educational, specialty, and subspecialty Nemazee and Hafez hospitals of the Shiraz University of Medical Science. Fathers in the intervention group learned how to support their wives and provide care for their premature newborns. The control group received the usual care. Mother’s stress and self-efficacy were measured using validated questionnaires.

**Results:**

Data analysis showed that the mean scores of mothers’ stress and self-efficacy from pre-intervention to post-intervention were significantly decreased and increased respectively in the intervention group (*p* <0.001). At the same time, there was no significant difference in the control group.

**Conclusion:**

When fathers are trained to support their wives and do so, it relieves the stress and improves the mothers’ self-efficacy, and has a direct effect on providing care to their premature newborns. Therefore, it is recommended that measures should be taken so that the fathers be present, participate in providing care, and support their wives and newborns in NICU.

**Trial registration:**

IRCT20171130037691N1.

## Introduction

Each year, approximately 15 million infants are born prematurely (i.e., less than 37 weeks of gestation) [1, 2]. In Iran, about 10% of the births are related to premature babies, and the country is one of the regions with a high prevalence of preterm delivery [3]. Prematurity of newborns is now the second leading cause of death in children under 5 years of age and the single most important direct cause of death in the critical first month of life [4]. Birth of premature newborns, admission in the NICU, and separation of newborns from families impose high stress on parents [5]. The stress level of parents with premature infants is a psychological hazard for parents [6]. Mothers with premature newborns hospitalized in NICU who are separated from their infant immediately after delivery have high stress, anxiety, and depression level. This negatively affects their thinking and decision-making processes, leading to increased stress and a lack of self-efficacy in the newborn’s care [7, 8]. The level of stress in mothers with premature newborns is higher than in fathers [9]. Mothers of premature infants have lots of stress and anxiety while their premature infants are in NICU. Therefore, there is an urgent need to relieve the mothers’ anxiety and stress [10]. These mothers’ stress and anxiety may lack appropriate newborn care ability [11, 12]. Parents attempt to support each other during the newborns’ hospitalization, but unfamiliar and critical situations prevent them from playing their role correctly [13]. The relationship between the parents indirectly affects their association with the newborn [14]. For example, realizing their husbands’ support and encouragement, mothers become more interested in performing their parental tasks [15, 16]. Those mothers not receiving support from the newborn father have less interaction with their infant than their peers who receive such support [15]. Some studies show that supporting mothers by their husbands could help overcome these problems [16–18]. This study aimed to train the fathers to support their wives and determine its effect on stress and self-efficacy in mothers of premature newborns hospitalized in the NICU.

## Methods

### Study design

This study had a quasi-experimental (pre-and post-intervention or before/after study) design with one experimental and control group and pre-and post-tests. We aimed to determine if training the fathers to support their wives would relieve stress and improve self-efficacy in the mothers of premature newborns hospitalized in the NICU. This design is considered to be of relatively high quality in the quasi-experimental study designs [19]. A quasi-experiment should be conducted when randomization is deemed not to be feasible [19–21]. In the current study, we selected a quasi-experimental design because randomization was not possible.

### Setting and participants

We have conducted this study in two hospitals affiliated with Shiraz University of Medical Sciences in the south of Iran. This study was carried out from 03/21/2018 to 09/11/2018. The research population included mothers with premature newborns hospitalized in NICU who were eligible for entering the study. Convenience sampling was done, and two groups of 45 and 30 parents were assigned to control and experimental groups, respectively. Sampling continued until the intended number of participants for each group was achieved.

The inclusion criteria for mothers and their newborns were premature newborns in the 32–37 weeks of pregnancy, lack of congenital anomaly in the newborn, lack of confrontation of mothers with other stressful events during the past 6 months, the outcome of a wanted pregnancy, lack of any record of using drugs effect on the mental health of the mothers, lowest elementary school literacy, a singleton infant, and lack of hospitalization record in NICU for their previous infants. Exclusion criteria were death or discharge of the newborn from the hospital during the study and lack of parents’ willingness to continue participation in the research.

The sample size was calculated according to Arshadi et al. [18], α = 0.05, β = 0.2, 1-β = 0.8, d = 14, *q*_1_ =0.4, *q*_2_ =0.6 and using following formula:

$$\mathrm{n}=\frac{\ {\left({z}_{\propto }+{z}_{\beta}\right)}^2\left(\ \frac{1}{q_1}+\frac{1}{q_2}\right)}{\frac{d^2}{s^2}}$$

Considering a 10% loss, the sample size was 75, of which 30 were placed in the intervention group and 45 in the control group. We sampled the two groups separately to prevent any diffusion of the intervention contents between the control and experimental groups. We primarily recruited participants for the control group and collected their data before implementing the intervention. After that, we recruited participants for the experimental group, delivered the intervention, and collected data from them at different time points.

### Measures

In this study, we used the Persian version of two questionnaires to collect the data: PSS (perceived stress scale) and PMP S-E (Perceived Maternal Parenting Self-Efficacy). PSS is a validated questionnaire for measuring the stress level of parents of premature newborns hospitalized in NICUs. The questionnaire includes 26 items in 3 dimensions: 5 related to NICU environment, 14 about appearance and behavior of infants and special treatments, and seven on parents’ relationship with newborns and playing their parental role. The items scoring are based on a 5-point Likert scale with a minimum score of zero and a maximum of 100. Higher scores indicate a higher degree of stress. The questionnaire is reliable through Cronbach’s alpha α = 0.87 for the whole questionnaire [22–24]. In a study performed by Beheshtipour et al. (2017), all subscales of the Persian version of PSS showed good internal consistency. (α = 0.77–0.86) [25].

The PMP S-E questionnaire, a tool has developed by Barnes and Adamson-Macedo with 20 declarative items, measure parents’ perceived feelings towards their self-efficacy. The instrument measures the parents’ perception of their capabilities in understanding and providing care to premature hospitalized newborns and their sensitivity towards various tasks and levels in playing the parental role for a premature newborn. The tool’s items were categorized as follows: 4 related to caring processes, 7 to motivational behaviors, 6 to the perception of behaviors and messages, and three related to situational behaviors. Scoring is within the range of 20 to 80, and high scores show higher levels of self-efficacy. The PMP S-E is a reliable scale (α = 0.91–0.96) [26]. Aliabadi et al. (2013) have used the questionnaire to evaluate the self-efficacy in mothers of hospitalized infants in several children’s hospitals in Iran. The above study was showed good reliability with a Cronbach’s alpha of 0.97 for the Persian version of PMP S-E [27].

### Procedure

The Ethics Committee of the Shiraz University of Medical Sciences approved this study (IR.SUMS.REC.1396.112). Then, after trial confirmation at the Iranian Registry of Clinical Trials (IRCT) with the code IRCT20171130037691N1, the researchers started the sampling.

All procedures performed in this study complied with the institutional and national research committee’s ethical standards and the 1964 Helsinki declaration and its later amendments or comparable ethical standards. We informed the Fathers and mothers who participated in the study about its purpose and procedure by face-to-face explanations, and we obtained written consent from them. First, sampling for the control group was done. Data were collected using the socio-demographic and the PSS and PMP S-E questionnaires through face-to-face interviews with mothers on the second day of the newborn’s hospitalization a week later. Participants in the control group received the usual training care. After completion of the control group, we started to select the intervention group. The socio-demographic, PSS, and PMP S-E questionnaires were filled out in the intervention group using the abovementioned method.

### Intervention

The experimental group’s fathers received an educational support program consisting of two 90-min face-to-face training sessions on the third and fourth day of the baby’s hospitalization. Contents of intervention were based on the nursing resources in the infant care field [28, 29] and provided using PowerPoint, videos, and an infant manikin model. Training sessions were held in the teaching rooms of Hafez and Nemazee Educational Health Centers. The materials provided to the intervention group were as follows:

#### First session

Welcoming, explaining the purpose, preterm birth, premature newborns’ status, and needs; explaining the NICU, health care staff, equipment, and usual therapeutic and nursing care provided in the ward; and answering participant questions.

#### Second session

Mothers’ physical and mental changes after childbirth, the role of fathers in providing support and care of the mother and premature newborn (having skin-to-skin contact between parents and their newborn, massage, feeding, assuming the proper position, and changing a diaper) and answering to participant questions. Also, for better learning of fathers, a booklet and two pamphlets about the care of premature newborns and mothers and familiarity with neonatal intensive care unit provided for them. After training, the fathers began caring about their infants and giving support to the mothers. All fathers completed the training courses without loss to follow-up. In the cases of any need for complementary information, they were provided with possible telephone contact or met the researcher. The usual care for the control group was included information about prematurity and the infants’ illness and giving a prognosis about the process of treatment, recovery of the baby, and introducing parents to the duties of the intensive care unit and staff.

### Statistical analysis

We used SPSS 21. to analyze the data. Descriptive data were analyzed through tables and charts (mean and standard deviations). Inferential statistics included chi-square, paired, and independent t-tests. PMP-SE has no dimension or category, but PSS can be categorized as very low (≤ 20), low [21–40], moderate (41–60), high (61–80), and very high-stress level (80 ≥). Statistical significance was considered *p* < 0.05.

## Results

**Fig. 1:**
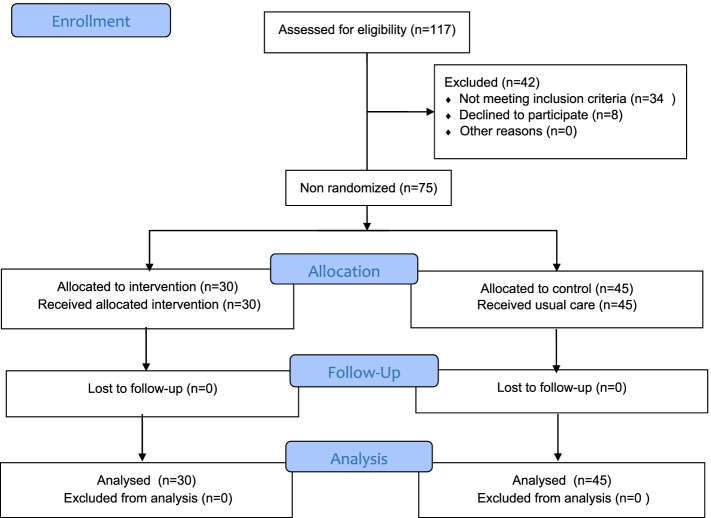
Flow chart of the study

In this study,117 participants were examined for eligibility, 42 were excluded due to 34 not meeting inclusion criteria, and eight declined to participate. Among 75 mothers under the study, 30 (40%) were in the intervention group and 45 (60%) in the control group (Fig. 1).

As shown in Table 1, both groups were homogeneous in terms of quantitative demographic variables (newborns’ birth weight, pregnancy age, mothers’ age, fathers’ age), and qualitative demographic variables (newborns’ gender, type of delivery, mothers’ education level, mother’s job, residence, being multiparous or nulliparous, miscarriage record, fathers’ education level, and father’s job). Statistical analysis showed no statistically significant difference (*p* > 0.05).

**Table 1:** Distribution of demographic variables

Group		Intervention	Control	*P* value
Variable				
Newborns’ gender	Male	14(46.7%)	23(51.1%)	0.70
	Female	16(53.3%)	22(48.9%)	
Type of delivery	Normal vaginal delivery (NVD)	12(40%)	13(28.9%)	0.31
	Cesarean section	18(60%)	32(71.1%)	
Mothers’ education level	Under the diploma	7(23.3%)	13(28.9%)	0.25
	Diploma and Bachelor	21(70%)	24(53.3%)	
	Master and Ph.D.	2(6.7%)	8(17.8%)	
Mother’s job	Employed	5(16.7%)	11(24.4%)	0.42
	Unemployed	25(83.3%)	34(75.6%)	
Being multiparous or nulliparous	Nulliparous	15(50%)	25(55.6%)	0.63
	Multiparous	15(50%)	20(44.4%)	
Abortion history	Yes	7(23.3%)	12(26.7%)	0.74
	No	23(76.7%)	33(73.3%)	
Fathers’ education level	Under the Diploma	5(16.7%)	2(4.4%)	0.06
	Diploma and Bachelor	17(56.7%)	36(80%)	
	Master and Ph.D.	8(26.7%)	7(15.6%)	
Father’s job	Employed	30(100%)	45(100%)	
	Unemployed	0	0	
Place of residence	Shiraz city	10(33.3%)	17(37.8%)	0.53
	City around	13(43.3%)	14(31.1%)	
	Rural	7(23.3%)	14(31.1%)	
Newborns’ birth weight (kg)		1892.66 ± 411.02	1772.88 ± 331.09	0.16
Pregnancy age (year)		34.36 ± 1.5	34.13 ± 1.45	0.50
Mothers’ age (year)		25.80 ± 5.65	24.53 ± 5.04	0.31
Fathers’ age (year)		29.33 ± 5.58	28.04 ± 4.22	0.25

As the results of Table 2, There was no significant difference between the stress mean scores in the intervention and control groups before the intervention (*p* = 0.13). There was a significant difference between the stress mean score in the intervention and control groups after the intervention (*p* < 0.001). In the control group, the stress mean scores before and after the intervention were not significantly different (*p* = 0.07). However, in the intervention group, the stress mean scores before and after the intervention were 72.96 ± 12.25 and 57.50 ± 6.29, and the difference was statistically significant (*p* < 0.001).

**Table 2:** Comparison of the mean stress before and after the intervention in both groups

Stress	Control	Intervention	Difference of means	95% CI	***p***-value
Group					
Before the intervention	68.28 ± 13.76	72.96 ± 12.25	−4.67	[−10.87, 1.51]	0.13
After the intervention	71.31 ± 9.78	57.50 ± 6.29	13.81	[9.78, 17.83]	< 0.001
***p***-value	0.115	< 0.001			

There was no significant difference between the self-efficacy mean score in the controls and the intervention group before the intervention (*p* = 0.057). There was a significant difference between the self-efficacy mean scores in the control and experimental groups after the intervention (*p* < 0.001). In the control group, the mean self-efficacy scores before and after the intervention were not significantly different (*p* = 0.115). However, in the intervention group before and after the intervention, the mean self-efficacy scores were 60.83 ± 4.77 and 70. 0 ± 4.82 respectively, with a statistically significant difference (p<0.001). The results are presented in Table 3.

**Table 3:** Comparison of the mean of self-efficacy before and after the intervention in both groups

Self-efficacy	Control	Intervention	Difference of means	95% CI	***p***-value
Group					
Before the intervention	63.66 ± 7.89	60.83 ± 4.77	2.833	[−0.37, 6.0]	0.057
After the intervention	60.95 ± 6.33	70.0 ± 4.82	−9.04	[−11.76, −6.32]	< 0.001
***p***-value	0.115	< 0.001			

**Fig. 2:**
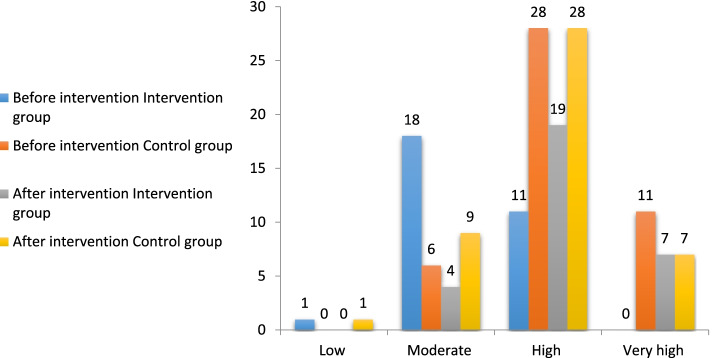
Comparison of the stress level by group (blue and orange for before intervention; gray and yellow for after intervention)

We used Wilcoxon signed ranks test to compare the two groups (Table 4, Fig. 2). The results showed that the stress levels before and after intervention in the studied groups were significantly different.

**Table 4:** Comparison of the study groups by the stress level

Total-Stress	N	Mean ± SD	Mean rank	Sum of rank	Test Statistics
Group					
Before the intervention	75	70.16 ± 13.30	33	95	***p*** = 0.009
After the intervention	75	65.78 ± 10.90	40	87	Z = −2.596

## Discussion

The present study aimed to train the fathers to support their wives and determine its effect on the mothers’ stress and self-efficacy scores in providing care to premature newborns hospitalized in NICU. The results showed that training fathers to support their wives reduced mothers’ stress and increased self-efficacy in the care of premature newborns. The differences between the mean scores of stress and self-efficacy in the control group before and after the intervention were not statistically significant. After the intervention, the mothers’ stress and self-efficacy scores decreased and increased in the intervention group. The difference observed in the intervention group before and after the intervention was statistically significant. Findings of numerous studies showed that interventions consisting of supportive (training-emotional) programs for parents of premature newborns reduced their stress and anxiety [28–30]. Hospitalization of the newborns resulted in stress in parents, especially mothers. Stress is a natural response to those events creating a feeling of threat, sadness, and lack of balance; however, long-term and high-stress levels can have physical and mental consequences [31, 32].

Arshadi et al. reported that family-based interventions reduce the mothers’ anxiety [18]. Also, Jafari et al. investigated the parents’ empowerment program’s impact on mothers’ anxiety and stress with premature newborns. They suggested that mothers’ stress and anxiety levels in the intervention group were less than those of the control group [33]. Shaw et al. reported that stress, anxiety, and depression reduced significantly after the supportive interventions in mothers with premature newborns hospitalized in NICU [34]. The results of these studies are consistent with those of our research. The study by Ong et al. indicated stressful conditions of the mothers whose premature infants were hospitalized at NICUs. They concluded that there is a crucial need to decrease their stress by interventional programs [10]. Meijssen et al. investigated the effect of behavioral evaluation of infants and intervention programs on mothers’ mental distress with premature newborns within the first 2 years of their lives. They found that mental distress didn’t significantly differ in the intervention and control groups [35]. The findings of these researchers are not consistent with those of the present study. The reasons for this discrepancy may be due to differences in the duration of the follow-up.

One of the best and most effective ways to prevent destruction resulting from the hospitalization of premature newborns is to provide caring intervention programs for parents [36]. Any mothers’ involvement in infant care can reduce stress because it relieves helplessness and increases their self-efficacy [37]. The relationship between parents and newborns is indirectly affected by that between the parents. For example, those mothers who realize the support and encouragement of their husbands become more interested in performing their parental tasks [15, 16]. Turan et al. investigated the effect of nursing intervention to relieve mothers’ stress in NICUs and found that parents experience high pressure when their infants are needed for intensive care; however, nursing interventions can decrease their stress [38]. Our study showed that a very high-stress level in the control group was more frequent than in the intervention group.

Hosseini et al. performed a clinical trial to determine the effect of relaxation training on improving mothers’ breastfeeding self-efficacy with premature newborns. Their results showed that the intervention increased the mothers’ breastfeeding self-efficacy [39]. Chan et al. studied the effect of self-efficacy-based breastfeeding training programs. They found that the training increased self-efficacy and breastfeeding duration through 6 months after childbirth [40]. In a study performed by Peyrovi et al. aiming at specifying the effect of mothers’ empowerment program on their readiness for premature infant care at the time of discharge from the hospital, it was shown that the status of practical and emotional readiness of mothers improved [41]. A study performed by Azmoudeh et al. showed that using self-efficacy promotion sources can improve feeling competent and the maternal role in mothers of Premature Newborns Hospitalized [42]. These results are consistent with those of the present study.

### Limitations

One study’s limitation was the relatively small sample size affecting medium and long-term outcomes for both mother and infant. In this regard, we tried not to lose any participants. Our study’s design was a non-randomized pre-posttest that could produce bias; then, we selected control and intervention groups sequentially from the two hospitals to reduce the bias.

## Conclusion

This study showed that mothers with premature newborns hospitalized in NICU experience a high level of stress. The main role of mothers in developing the hospitalized premature newborn, taking some measures to reduce their stress and increasing their self-efficacy, seems necessary.

In the present study, training fathers’ intervention to support wives reduced their stress and increased their self-efficacy. Thus, taking advantage of fathers’ participation in NICU and training them as early intervention NICU purposively is suggested, considering their valuable role in supporting the mothers.

## Data Availability

The dataset used and analyzed during the current study is available from the corresponding authors upon official request.

## Abbreviations

NICU: Neonatal Intensive Care Unit
PSS: Perceived Stress Scale
PMP S-E: Perceived Maternal Parenting Self-Efficacy
